# Synthesizinge a novel Zr2Al-GNS MAX phase ceramic with superior electrical properties using pressureless sintering technique

**DOI:** 10.55730/1300-0527.3577

**Published:** 2023-07-04

**Authors:** Dumooa R. HUSSEIN, Khalid K. ABBAS, Ahmed M.H. Abdulkadhim AL-GHABAN

**Affiliations:** Department of Materials Engineering, University of Technology, P.O. Box 19006, Baghdad, Iraq

**Keywords:** MAX phase, nanographene sheet, pressureless sintering, Zr_2_AlC ceramic, powder technology

## Abstract

A unique Zr_2_Al-GNS MAX phase ceramic supported nanographene sheet was prepared using a cost-effective pressureless sintering technique under relatively low temperature. An experimental investigation was conducted to explore the lattice parameters using different temperatures, such as 1000, 1150, and 1300 °C. To characterize the crystal structure of the MAX phase ceramic, X-ray diffraction, field emission scanning electron microscopy imaging, energy-dispersive X-ray spectroscopy (EDX), high-resolution transmission electron microscopy (HRTEM), and selected area diffraction (SAED) were utilized. The results revealed that the pressureless sintering technique was successfully utilized to synthesize the Zr_2_Al-GNS MAX phase ceramic under 1150 °C with a low impurity ratio of secondary phases such as Zr_3_AL_2_, Zr_3_AL_5_, and ZrC components. The high percentage of the Zr_2_Al-GNS MAX phase ceramic was obtained at 49.0% at 1150 °C compared with different temperatures. The BET surface area (S_BET_), pore volume, and pore size were also investigated. The S_BET_ of the prepared Zr_2_Al-GNS MAX phase was increased to 30% using graphene nanosheet, while the porosity was highly decreased to 8% from its original value. The electrical properties were also studied in this research for potential applications, such as the absolute value of impedance (Z), absolute value of admittance (Y), induction (L), capacitance (C), resistance (R), conductance (G), susceptibility (B), and phase angle (ϴ). It was found that the capacitance and the phase angle were improved using the prepared Zr_2_Al-GNS MAX phase ceramic, depending on the frequencies. The results presented here may facilitate the improvements in the features of the MAX phase type of Zr_2_Al-GNS-enhanced one-layer nanographene sheet for electrical applications ceramic.

## 1. Introduction

**Scheme 1:**
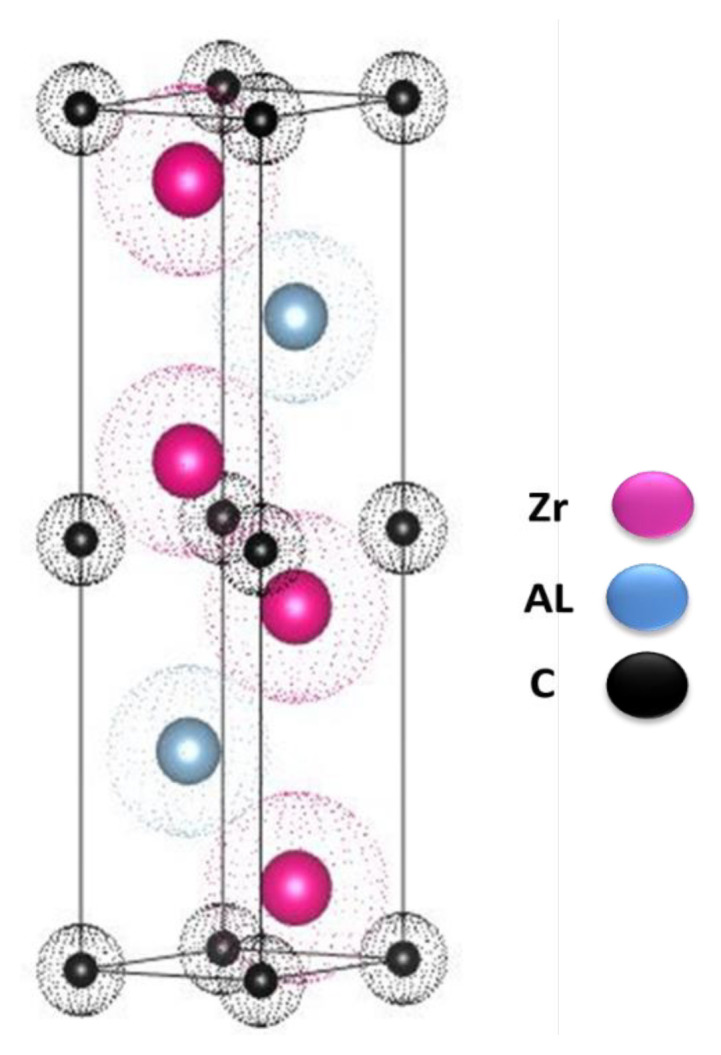
The Zr_2_ALC MAX phase ceramic structure.

The difficulty in synthesizing high-purity ceramic MAX phase at low temperatures is a major issue recently. The MAX phases can be defined as a family of carbides and nitrides with a hexagonal structure (space group P6_3_/mmc; No.194) in the general formula of M*_n_*_+1_AX*_n_* with n being an integer, M being a transition metal, A being a group of 13 to16 elements. X is C/N/B [1–5]. Scheme 1 shows the unique structure of the Zr_2_ALC MAX phase.

MAX phases have been classified into the following groups based on the value of n: M_2_AX (211 phase), M_3_AX_2_ (312 phase), M_4_AX_3_ (413 phase), M_5_AX_4_ (514 phase), M_6_AX5 (615 phase), M_7_AX_6_ (716 phase) [6], hybrid MAX phases 523 phase (211 phase +312 phase) and 725 phase (413 phase + 514 phase) [7], M_2_AB_2_(212 phase) and M3AB4 (314phase) boride MAX phases [8–10]. More than half of the discovered MAX phases, with *n*=1, were first reported in the late 1960s by Nowotny et al. [11,12]. The carbides with a general formula of M_2_XC are called H-phases, in which X is the B-group from l to 3 elements. The H-phases are ternary layered compounds that the hexagonal B-group elements net is separated as layers from the edge-shared transition metal carbide octahedral structure. Indeed, 40 H-phases have been found containing transition metals such as Al, Ga, In, Tl, Sn, Ge, Pb, S, As, Cd, Zn, and P [13]. For example, Lapauw and coworkers synthesized Zr_2_AlC and Zr_3_AlC_2_ compounds experimentally, which are considered the first MAX phases in the Zr-Al-C system [14]. A new family of 2D transition-metal carbides/nitrides known as MXenes was discovered in 2011 by the synthesized 2D titanium carbide (Ti_3_C_2_) selecting aluminum to be etched from the MAX phase of Ti_3_AlC_2_ (312). MXenes are promising as a wide range of applications such as catalysis because of their exceptional characteristics such as large specific surface area. Including new novel layered in the MAX phase compounds has received a lot of attention recently, in particular because of the possible applications of MXenes. It is possible to make a straightforward attempt by substituting boron atoms for C/N atoms. No results using this method have been obtained. The M–B bond is weaker than the M–C or M–N bonds, suggesting that the MAX phase containing boron is less stable thermodynamically than its equivalents in the carbide or nitride states. Ade and Hillebrecht reported that the MAB phases, a new family of layered transition-metal borides (M, A, and B) can denote an early transition metal such as IIIA or IVA group element, and boron, respectively). Kota et al. have evaluated the synthetic procedures, crystal structure, chemical bonding, and distinguishing the characteristics of MAB phases. Several theoretical and experimental findings show that MAB phases can exfoliate into 2D transition-metal borides (MBenes) [10]. The synthesized MAX phase could be more than 150 types [15,16].

There are many fundamental techniques to synthesize the MAX phase structure, such as the physical vapor deposition (PVD) technique, solid-state reactions, and the molten process. One of the main techniques is the solid state reactions method, including pressureless synthesis (PLS), self-propagating high-temperature synthesis (SHS), hot-pressing (HP), spark plasma sintering (SPS), and the microwave (MW) process. Generally, the MAX phases are relatively easy to synthesize, but producing high yields (>95 wt%) is an exception to achieve the MAX phase, which coexists with other thermodynamically stable phases, for instance, carbides or nitrides, as well as intermetallic compounds [17].

The MAX phase is a unique combination of ceramic and metallic properties producing the *n* “ceramic” MX layer (s), which is interleaved by an A “metallic” plane. This new structure includes excellent properties such as high thermal shock resistance, high stiffness, good thermal and electrical conductivity, resistance to corrosion, and antioxidation capability [18,19]. Therefore, the application of the MAX phases can be potentially used as heating elements, armor, nuclear industries, aerospace, automotive, defense, and medical applications [11,12]. To give more examples, the ceramic MAX phases of Ti_2_AlC, Ti_3_AlC_2_, and Cr_2_AlC have superior properties, such as self-healing properties [20–22], as well as reversible deformation, which have attracted scientists worldwide. The ceramic MAX phase can also achieve a high melting point, excellent hardness, and superior thermomechanical/thermochemical properties. These properties can lead to a high dielectric constant with attractive optical characteristics obtaining ideal optoelectronic applications [23,24]. More than seventy advanced MAX phases have been recently synthesized, and it is expected that more innovative MAX phase structures can be achieved potentially. One of these structures is Zr_2_AC MAX phases which contain A = Al, Si, P, S, Ga, Ge, As, Cd, In, Sn, Tl, and Pb [25]. Particularly, the ceramic MAX phase contains Al-element, a unique combination of ceramic and metal properties, which can be applied in anticorrosion and resistant oxidation when utilized in high-temperature environmental applications. Such MAX phases (Zr_2_AC) can be expected as prospective materials for accident-tolerant fuel cladding in light water reactors, where the materials are commonly exposed to high mechanical and thermal loads with strong neutron irradiation and high oxidation regions [25]. The Zr-based ceramic MAX phase is considered useful in nuclear environmental applications, particularly the Zr_2_AlC. The high neutron transparency of the Zr element offers highly desirable reactor neutronics, thereby reducing the fuel consumption in the nuclear reactor. The presence of the Al element in the ceramic MAX phase can assist in forming a protective oxide layer to prevent the high-temperature steam oxidation process [26]. Because of the narrow cross section of the Zr element in neutrons applications, the Zr-Al-C MAX phases ceramic, such as Zr_3_AlC_2_ and Zr_2_AlC, are superior candidates for using in fuel cladding coatings [27,28]. Therefore, a simple, cost-effective technique with uncomplicated equipment under low temperature preparing a high-purity ceramic MAX phase is in urgent demand.

Numerous studies have attempted to synthesize the MAX phases; for example, Lapauw and coworkers investigated the effect of synthesis temperature on the structure of the Zr_2_AlC MAX phases. They claimed that the hot pressing sintering technique (HPS) successfully synthesized the ceramic MAX phase of the Zr_2_AlC type at a temperature range of 1475–1575 °C, obtaining an impure ZrC component as a secondary phase. Additionally, they revealed that the synthesis temperature was 1525 °C with 67 wt% yield of the Zr_2_AlC MAX phase and 33 wt% of impure ZrC_x_ component. The challenges encountered in the research made it significantly difficult to obtain the Zr_2_AlC MAX phase at low temperature with high purity [29]. A previous study by Haemers and colleagues proposed that the ceramic MAX phase Zr_2_AlC can also be prepared using the hot press sintering (HPS) technique, and they successfully reduced the impure quantities of Zr_3_AlC_2_ and ZrCx components. The synthesized ratio was 2Zr: 0.8Al: 1.2C with a sintering temperature ranging from 1525 to 1575 °C. Additional efforts by the same researchers attempted to synthesize ceramic MAX phase Zr_2_AlC using a pressureless sintering technique (PLS). The samples were sintered at 1900 °C for 10 min before being held at 1600 °C for 1 h, 1450 °C for 1 h, 1300 °C and 1150 °C for 10 h under the atmosphere of the argon gas. The problem faced by researchers in this method is the formation of a new phase which is the ZrC component at high temperatures with Zr-Al alloy components. The researchers also assumed that the PLS method is considered to mix the zirconium with other materials such as Cr, Mo, or Ti to form (Zr_x_M_1-x_)_2_AlC compounds. However, none of these compounds contains the Zr-based MAX phase. Based on this research, pressureless sintering is not anticipated to be able to produce the Zr_2_AlC MAX phase with high purity [30].

To obtain high interfere atoms in the ceramic MAX phases instead of the carbides, it is possible to use a nanocarbon material to facilitate the manufacturing process with high purity at low temperature. One of these nanomaterials is graphene in the form of a single layer which can form one unit of the MAX phase with excellent network distribution, obtaining intermolecular overlap to achieve new class specifications stronger than the normal ceramic MAX phase. Graphene is a flat monolayer of carbon atoms firmly packed into a two-dimensional (2D) honeycomb lattice. In addition to its unique structure, graphene has a number of interesting characteristics, such as thermal conductivity and mechanical stiffness equivalent to graphite’s original values, and individual graphenes are reported to have good electrical transport capabilities, optical, magnetic, thermal, and mechanical properties, as well as a large specific surface area [31,32]. These characteristics have great potential applications, including nanoelectronics, sensors, transistors, and batteries [31]. Graphene is covalently bonded with a two-dimensional building block of sp2 hybridized carbon and allotropes of other carbon-based compounds [33]. Graphene as nanosheets can improve the ceramic MAX phase structure and its properties. To the best of the authors’ knowledge, no report has been found so far using graphene nanosheets, including into the ceramic MAX phase. The graphene can be prepared as nanosheets using different techniques, such as epitaxial growth on SiC, chemical vapor deposition (CVD) on metal substrates, pyrolysis of polymer films, exfoliation of graphite in organic solvents, and electrochemical reduction of exfoliated graphite oxide (GO) [34,35].

This study utilized the powder metallurgy technique to synthesize the ceramic MAX phases using cold pressing and then the PLS technique. This simple pathway reduces a lot of obstacles, such as the costs and impurities increasing the efficiency of the ceramic MAX phase, and opening a new prospective for developing advanced ceramic MAX phases materials realistically. The PLS technique is a simple, cost-effective, precursor-flexible method that is scalable to large scales and can be adjusted to yield a high purity of the ceramic MAX phases such as Zr_2_AlC. High purity MAX phase can be obtained using the PLS process up to 92%–98% with porous dense pellets [17,36]. The challenges of this process are that it produces fully dense components with fewer impurities [37]; the aluminum in the structure of the ceramic MAX phase is highly volatile, and the element ratio is a critical factor that must be firmly controlled. To solve this problem, the nanographene sheet network is incorporated into the MAX phases ceramic to produce a high-purity Zr_2_Al-graphene nanosheet (Zr_2_Al-GNS). The structure of the prepared MAX phase ceramic of the Zr_2_Al-GNS was characterized, and the electrical properties were addressed.

### 1.2. Materials

Graphite flakes with a particle size of 500 μm were purchased from Laboratory Chemicals, India. Formic acid (HCOOH, conc. 85%) was supplied by LUXI (product specification GB/T2093-2011). ZrH_2_ (>99.9%, 325 mesh) was purchased from Luoyang Tongrun Info Technology Co., Ltd. High purity AL (>99%, 325 mesh) was acquired from HIMEDIA Company.

## 2. Synthesis of the ceramic MAX phase

### 2.1. Preparation of graphene

Graphite flakes were ground to a particle size of 37 μm using grinding (Silver Crest Power grinding SC-1880), and then a round-bottom flask was filled with 1 g of graphite containing 50 mL of formic acid. The mixture was ultrasonicated (power sonication 410) at room temperature to produce multilayer graphene nanosheets (GNSs/HCOOH). After a while, the multilayer GNPs were well-dispersed in the solution to obtain the graphene nanosheet (GNS) as layers. The resultant was filtered using a Whatman filter paper with the size of 20–25 μm and washed well with dilute acetone solution, and then a few unexfoliated graphite flakes were removed from the bottom of the flask. Finally, the GNS was dried in a vacuum oven overnight to obtain graphene nanosheet [38], which is ready to be the main material to prepare the ceramic MAX phase.

### 2.2. Preparation of the MAX phases ceramic (Zr_2_Al-GNS and the Zr_2_AlC)

**Scheme 2:**
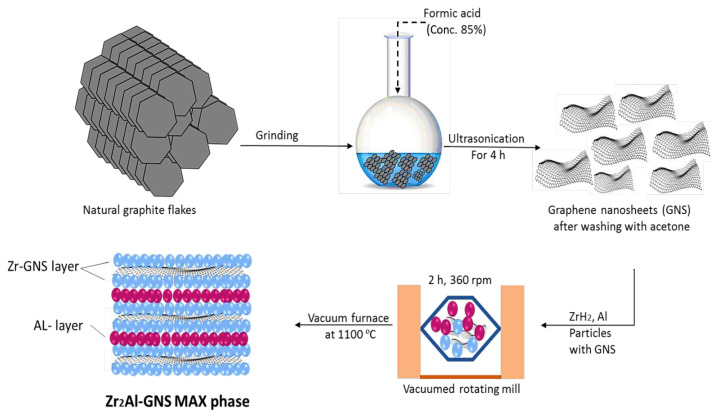
Illustration of the preparation of the Zr_2_Al-GNS ceramic MAX phase.

In this work, firstly, the Zr_2_Al-GNS was prepared using ZrH_2_, aluminum, and the prepared nanographene sheet as reactants. The particles were mixed (stoichiometry ratio of (2:1:1.2) Zr: Al: GNS) in a vacuum rotating mill (Planetary Ball Mill 4X500ml-Lubrication Free, Vacuum & Inert Gas Compatible) containing balls made from ZrO_2_ material (size of 5 mm). The ZrO_2_ balls were utilized to homogenize the mixing and break up the soft agglomeration of the samples to obtain a homogenous mixture. Figures S1 and S2 in the supplementary material illustrate no agglomeration in the samples. Importantly, the reactants were mixed in a vacuum container to minimize the oxidation reaction. The jars were sealed and spun for 2 h at 360 rpm in a vacuum rotating mill. After that, the powder mixture was placed into 25 mm inner-diameter stainless steel die and cold-compressed at 400 MPa at room temperature (MEGA KC 50 manual hydraulic press, made in Spin). The sample is then heated at a rate of 20 °C/min in a vacuum tubular furnace at 1000 °C, 1150°C, and 1300 °C (furnace type, MTI Corporation GLS 1500X). Secondly, this procedure was repeated using the graphite particles instead of GNS to obtain the ceramic MAX-phase for comparing, as displayed in Scheme 2. The achieved samples were characterized by utilizing different characterization apparatuses.

### 2.3. Characterization

The powder samples were analyzed using a Lab XRD-6000, a Shimadzu X-ray diffractometer set at 40 mA and 40 Kv with Cu-K radiation (λ = 1.5418) at the 2ϴ = range of 10–80 at a scanning rate of 28 min^–1^. Crystalline phase determination was done with the assistance of X’Pert High Score Plus software using the ICDD (International Centre for Diffraction Data) database. A current of 10 A was used at a 5 Kv acceleration voltage. Prior to the analysis performance, the samples were put on a carbon tap surface with a very thin platinum coating. Furthermore, field emission scanning electron microscopy was done to analyze the synthesized Zr_2_AlC and the Zr_2_Al-GNS ceramic MAX, and the elemental composition of the synthesized MAX phase was examined by EDX (energy-dispersive X-ray spectroscopy). Field emission scanning electron microscopy imaging (FESEM) device: (TESCAN Mira^3,^ made by the Czech Republic, Iran University Moktabar, Tehran, Iran) and the EDX device are the same as the FSEM device and only detector change (SE). High-resolution transmission electron microscopy (HRTEM) and selected area diffraction SAED (X the X-MAX^N^ 80^T^ instrument from Oxford Instruments Nano Analysis, OHSAS18001, which is located at Iran University Moktabar in Tehran, Iran) were utilized. The BET surface area analysis was carried out using the (BEL-BELSORP MINI X instrument at Iran University Moktabar Ltd. Co., Tehran, Iran). The Brunnauer-Emmett-Teller method was used to calculate the specific surface area using N_2_ adsorption-desorption isotherm equations. The Barrett–Joyner–Halenda (BJH) model was used to derive the pore size distributions from the adsorption branches of isotherms at 77 ± 0.5 °K in liquid nitrogen. Prior to measuring the BET surface area, the sample vessels were loaded with 0.5–1.0 g and degassed at a high temperature of up to 200 °C overnight with an evacuation pressure of 50 mTorr.

## 3. Electrical properties

Electrical resistance (voltage-current curve) was carried out using an l-V measurement device (NPRAGA Company). The absolute value of impedance (Z), absolute value of admittance (Y), induction (L), capacitance (C), resistance (R), conductance (G), Susceptance (B), phase angle (ϴ), quality factor(Q), dissipation factor (D), and reactance (X) were investigated.

## 4. Results and discussion

### 4.1 X-ray diffraction (XRD)

The XRD patterns of the prepared ceramic MAX phases, including the Zr_2_AlC and Zr_2_Al-GNS, were characterized at various temperatures, such as 1000 °C, 1150 °C, and 1300 °C. The phase quantification was conducted using Equation 1, as reported elsewhere [39,40]. In this equation, the phase ratio is equal to the ratio between *I_x_* that represented the integrated area of the most intense peak of the phase, and *I_t_* is the total sum of the integrated area of the most intense peak for all representative phases (Zr_2_AlC, Zr_2_Al-GNS, Zr_3_Al_2_ & Zr_5_Al_3_ and ZrC). The least-squares method was used to compute the integrated areas [39,40].


Eq. 1 $$Phase\;(\%)=\frac{I_{x}}{I_{t}}$$[
39,
40]

**Figure 1:**
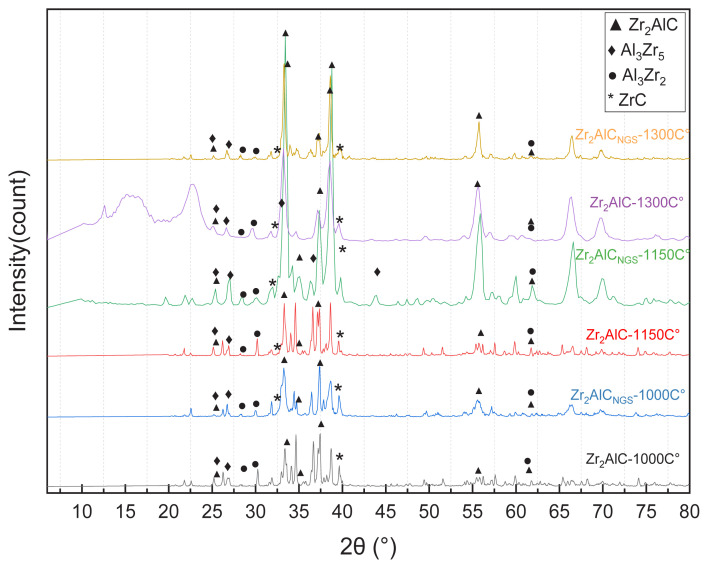
The Zr_2_Al-GNS and Zr_2_AlC MAX phases XRD pattern at sintering temperatures of 1000 °C, 1150 °C, and 1300 °C.

According to JCPDS cards numbers 98-005-8232 and 98-060-9718, the intermetallic Zr_3_Al_2_ and Zr_5_Al_3_ compounds appeared when using the sintering temperature of 1000 °C for both Zr_2_AlC and Zr_2_Al-GNS, respectively. The binary carbide phase ZrC also appeared (JCPDS card number 98-061-9173), while the Zr_2_AlC MAX phase was detected as a secondary phase confirming low crystallinity with high impurities were discovered. At 1150 °C, the XRD pattern of the prepared Zr_2_AlC revealed high impurities with low crystallinity approaching new phases such as Zr_3_Al_2_, Zr_5_Al_3_, and ZrC. In contrast, the XRD intensity peaks of the synthesized Zr_2_Al-GNS revealed that few impurities with high crystallinity phases, such as ZrC and Zr_3_Al_2_ compounds, appeared. Interestingly, using a single layer of graphene nanosheet in the prepared Zr_2_Al-GNS improved the crystallinity, reducing the impurities simultaneously due to the nano graphene sheet having a high specific surface area compared with graphite, which increases the reactivity of the sintering reaction. This has coincided with Boch and Niepce’s study [41]. The increase in sintering reactivity can be attributed to increased bonding between atoms because of the superior high surface area of graphene nanosheet and small particle size, which increases diffusivity through the sintering process. The nano graphene sheet structure increases from its ability to form ternary carbide. All of these characteristics increase the ternary carbide MAX phase’s ability to be formed. As illustrated in Figure 1, low intensity diffraction peaks of the synthesized Zr_2_AlC and Zr_2_Al-GNS were obtained at 1300 °C with high impurity phases such as Zr_5_Al_3_ and ZrC.

The crystallinity percentage was also calculated according to Equation 1. The high percentage of the Zr_2_Al-GNS MAX phase ceramic was obtained to be 49 wt% at 1150 °C compared with different temperatures. Table 1 and Figure S3 in the supplementary material show the prepared samples’ crystallinity phase with the impurities’ percentage. The following point can explain the results of the XRD test:

- The main phases are Zr_2_AlC, Zr_3_Al_2_, Zr_5_Al_3_, and ZrC with other phases in very small quantities, which did not appear in all sintering temperatures.
- The Zr_3_Al_2_ intermetallic compound is possibly formed in a high ratio at low temperatures than 1000 °C and decreases at high temperature of 1300 °C compared with Zr_5_Al_3_ intermetallic compound.
- The Zr_5_Al_3_ increases at high temperatures due to the Zr_5_Al_3_ (more complex compound) requiring a higher temperature to be formed than the Zr_3_Al_2_ intermetallic compound.
- The ZrC carbide was formed at a low temperature of 1000 °C, and it increases at high temperatures of 1300 °C due to its dissociated unstable MAX phase at high temperatures of 1300 °C to convert to metal carbides (ZrC_X_) and A element [13,42].
- At 1150 °C, impurities or secondary phases of Zr_3_Al_2_, Zr_5_Al_3_, and ZrC may decrease, confirming that the main phase of Zr_2_AlC MAX phase appeared. Furthermore, the MAX phase synthesis was unstable, so the synthesized ternary carbides can be found to dissociate into the transition metal carbide MC_x_ and the A-group element at a high-temperature range, as found by El-Raghy et al. and Barsoum et al. [13,42] in Zr_2_SnC and Zr_2_PbC MAX phases at a temperature range of 1250 ± 1390 °C.

Importantly, the main peak of the Zr_2_Al-GNS MAX phase ceramic was shifted from 37.3° to 25.15° due to the lattice of the MAX phase was expanded [43–46], indicating an elemental overlap occurred with the single layer of graphene nanosheet. The crystallite size was calculated using the Debye-Scherrer equation Equation 2 [47]:


Eq. (2) $$D=\frac{K\lambda }{\beta \;COS\;\theta }$$[
47]

where D is the size of the coherent diffraction domain in nm, λ = 0.15406 nm represents the wavelength of the applied X-ray source, *β* = the reflection width (2θ) FWHM (radians), θ = the Bragg angle, and K = 0.9 the shaped constant, respectively [47]. Table 2 illustrates the crystal diameter of the prepared samples. The crystal diameter of the synthesized Zr_2_Al-GNS at 1150 °C was 24.20 nm using nanographene sheet, which is higher than graphite, causing the crystals to expand notably. Therefore, increasing the MAX phase’s crystal size was achieved by utilizing nanographene sheet.

The lattice parameters of the MAX phases compared with those of previous studies were also calculated in the range of: 3.1 < a < 3.4 and 13.6 < c < 14.7 A ° [48]. For comparison, the lattice values were listed from the literature. The lattice parameters coincided with the experimental data in this work, as illustrated in Table 3.

### 4.2. FESEM

**Figure 2:**
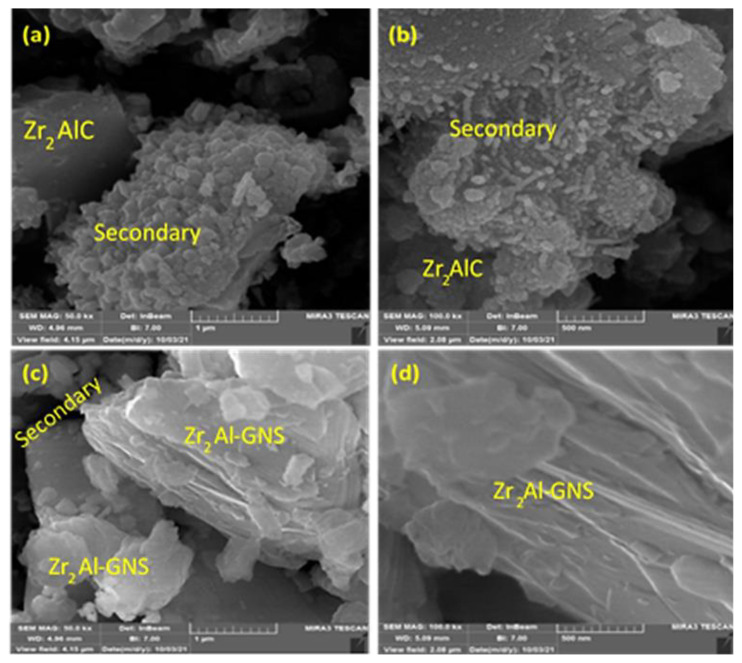
The FESEM images of the Zr_2_AlC MAX phase (a, b) and the Zr_2_Al-GNS phase (c, d).

Field emission scanning electron microscopy was done to analyze the synthesized Zr_2_AlC and the Zr_2_Al-GNS MAX phases ceramic at 1150 °C, as shown in Figures 2a–2c. In this test, the laminated layers of the prepared Zr_2_AlC MAX phase have been transformed inconsistently to the disjointed layers with each other because of the lack of interconnection between these multilayers or laminates as well as the presence of a significant degree of impurity content as illustrated in Figures 2a and 2b. Additionally, Figures 2a and 2b show secondary phases such as Zr_3_Al_2_ & Zr_5_Al_3_ and ZrC like cluster with small quantities of pure phase.

In contrast, the laminated layers of the prepared Zr_2_Al-GNS MAX phase ceramic have well interconnections due to the nanographene sheet network that conjugated the MAX phase layers perfectly with small fragments of impurities (Zr_3_Al_2_ & Zr_5_Al_3_ and ZrC) as displayed in Figures 2c and 2d. The pressureless sintering process (PLS) has been verified to have successfully synthesized the Zr_2_Al-GNS MAX phase ceramic with high purity and density of the nanolaminated-layers of MAX phase, obtaining that the atoms of carbon in the graphene nanosheets (GNS) were well bonding one with each other as well as tightly bonding with the Zr forming carbide layers of ZrC separated by AL layers in Zr_2_Al-GNS MAX phase ceramic crystal. These crystals are remaining conjunction with other crystals with assistance by the GNS material.

**Figure 3:**
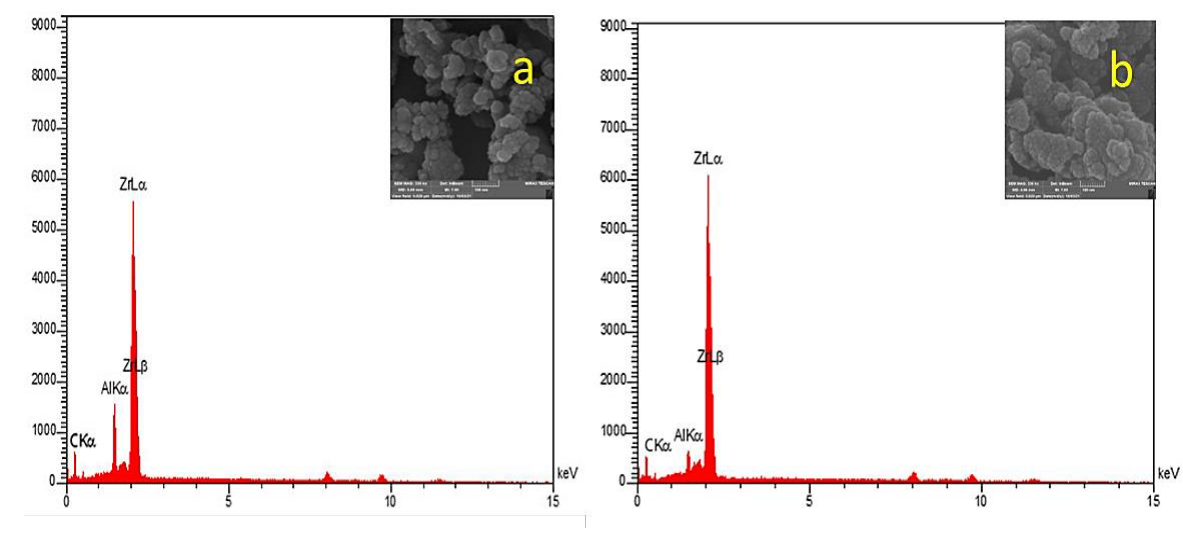
(a, b) Energy dispersive X-ray spectrometry (EDX) analysis for (a) Zr_2_Al-GNS ceramic MAX phase and (b) Zr_2_AlC MAX phase, respectively.

At 1150 °C, the prepared Zr_2_AlC and Zr_2_Al-GNS MAX phases ceramic were also investigated using energy dispersive X-ray spectrometry (EDX). The weight ratios and atomic ratios of Zr_2_AlC and Zr_2_Al-GNS MAX phase ceramic are shown in Table 4. In this analysis, the Zr, Al, and C elements were well-distributed in the Zr_2_AlC and Zr_2_Al-GNS MAX phases, as seen in Figures 3a and 3b. However, the intensity peaks of the ceramic MAX phase of the Zr_2_Al-GNS were relatively higher than the synthesized Zr_2_AlC MAX phase ceramic, confirming an excellent elemental distribution when using the nanographene sheet in between the layers. The Zr_2_Al-GNS coincided with the type of 211 MAX phase, as displayed in Figure 3b.

Interestingly, the atomic weight of the aluminum element in the structure of the Zr_2_AlC MAX phase was 12.4%. In comparison, the atomic weight of the aluminum element in the structure of the Zr_2_Al-GNS MAX phase ceramic was increased to 20.4%, conserving its original value as illustrated in Table 4. This phenomenon is due to the sublimation process, which occurred during the sintering of Zr_2_AlC MAX phase specimens. This is explained by that the availability of the graphene nanosheet prevented the Al element from escaping easily out of the Zr_2_Al-GNS MAX phase’s structure, therefore supporting and promoting its structure [43,52–54]. It is expected that the GNS can react with aluminum instantaneously, forming Al_4_C_3_ compound, which is essential to build up the MAX phase. This phenomenon is similar to that in the stable MAX phase of Fe_2_AlC [55]. The reaction of the Al_4_C_3_ compound is illustrated in Equation 3:


Eq. (3) $$\text{ZrC}+{\text{Al}}_{4}{\text{C}}_{3}+7\;\text{Zr }\;\;4{\text{Zr}}_{2}\text{AlC}$$[
55]

Another plausible explanation is that the GNS can be instantaneously reacted with zirconium forming ZrC a carbide, which reacts with Zr_2_Al_3_ intermetallic compound forming Zr_2_Al-GNS MAX phase as suggested by Haemers [29]. Equation 4 shows the reaction between ZrC carbide and Zr_2_Al_3_ intermetallic compound.


Eq. (4) $${\text{Zr}}_{2}{\text{Al}}_{3}+4{\text{ZrC}}_{0.75}\to 3{\text{Zr}}_{2}\text{AlC}$$[
29]

Therefore, incorporating the graphene nanosheet (GNS) in the ceramic MAX phase was boosted from layers to be highly integrated.

**Figure 4:**
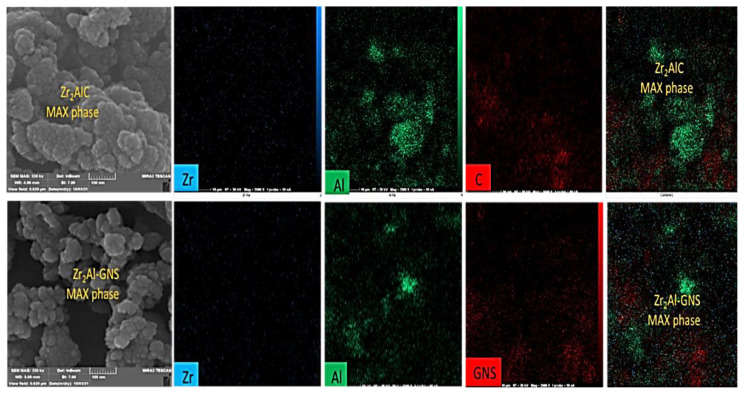
The EDX mapping for the Zr_2_AlC and Zr_2_Al-GNS MAX phases, respectively.

The EDX mapping was also studied for the prepared Zr_2_AlC and the Zr_2_Al-GNS MAX phases for more evidence. From Figure 4, a high density with well-distribution elements was obtained in the Zr_2_Al-GNS MAX phase ceramic structure. In contrast, less dense elements in the Zr_2_AlC MAX phase ceramic were achieved, as displayed in the EDX mapping photos as depicted in Figure 4, indicating that the GNS single layer was highly affected by the MAX phase to be an excellent bonding with the Zr elements by (Zr-C) form carbide layers as well as bonding this Zr-C carbides layers with pure aluminum layers (carbides layers separated by AL layers). Additionally, it can be noticed that the purity of the Zr_2_Al-GNS MAX phase ceramic was increased using the nanographene sheet instead of graphite flakes.

### 4.3. HRTEM and the SAED pattern

**Figure 5:**
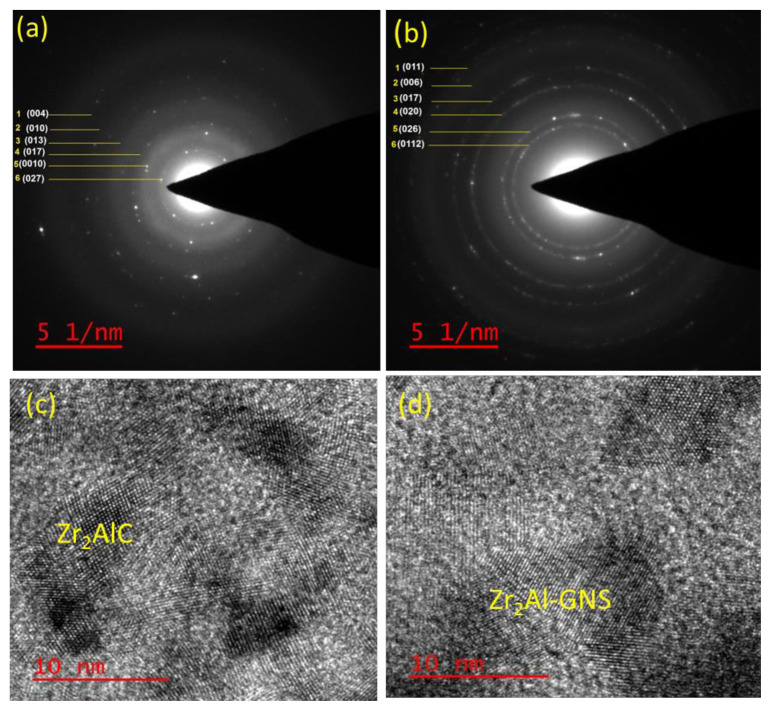
The HRTEM images (c,d) and (a, b) of the SAED pattern of Zr_2_AlC and the Zr_2_Al-GNS MAX phases, respectively.

The HRTEM and the SAED pattern of the prepared Zr_2_AlC and the Zr_2_Al-GNS MAX phases ceramic were investigated. These tests were done to confirm that the multilayer is stacking in the atomic structure of the prepared Zr_2_AlC and Zr_2_Al-GNS MAX phases, and the crystal structure type was identified as illustrated in Figures 5a–5d. Figures 5a and 5b shows the polycrystalline structure and the SAED pattern for the Zr_2_AlC and the Zr_2_Al-GNS MAX phases supported by HRTEM photos Figures 5c and 5d. According to Figure 5a, the weak bonding between zirconium and carbon atoms form carbide along with aluminum layers generating a Zr_2_AlC MAX phase with an unfavorable and poor atomic layers structure staking. Table S1 in the supplementary material presents the d-spacing and (*hkl*) values of the Zr_2_AlC and Zr_2_Al-GNS MAX phases estimated from the SAED pattern. The *hkl* values for the Zr_2_AlC and Zr_2_Al-GNS MAX phases were calculated using the SAED pattern, as shown in Figures 5a and 5b; while the prepared Zr_2_Al-GNS was successfully achieved using GNS layers with good circles layers’ arrangements. As illustrated in Figures 5c and 5d, the polycrystalline structure is the dominant phase than the single crystalline structure.

### 4.4. The BET surface area (S_BET_)

**Figure 6:**
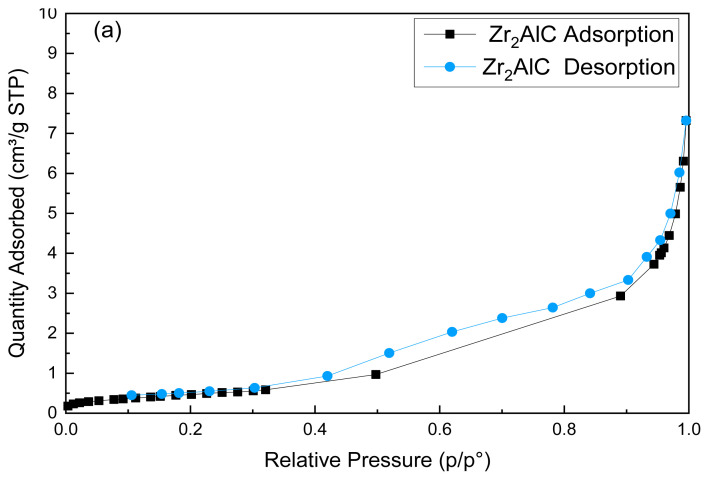
(a,b) The adsorption/desorption isotherm of (a) the Zr_2_AlC MAX phase ceramic and (b) the Zr_2_Al-GNS MAX phase ceramic.

**Figure 7:**
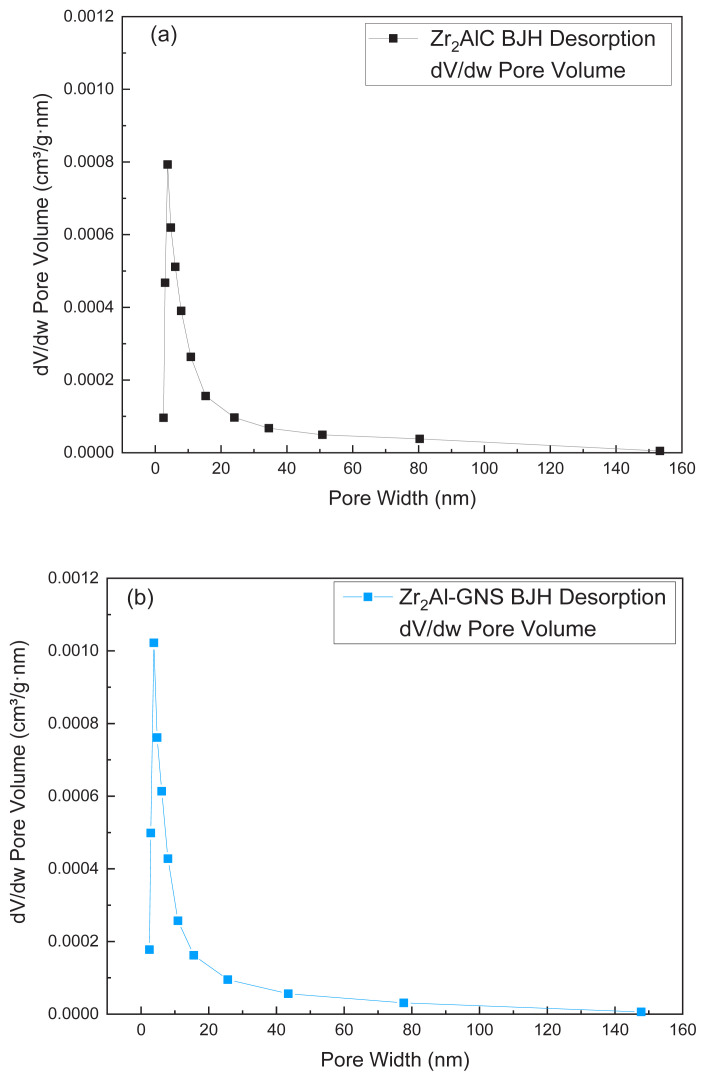
(a,b) The pore size distribution of (a) the Zr_2_AlC MAX phase ceramic and (b) the Zr_2_Al-GNS MAX phase ceramic.

The high surface area plays a vital role in providing multiple effective sites supporting the atomic layers to be highly bonding with each other [41,56]. Based on this, the presence of graphene enhanced the surface area of the ceramic MAX phase. The S_BET_ and pore size distribution of the Zr_2_AlC and Zr_2_Al-GNS MAX phases ceramic were illustrated in Figures 6a and 6b and Figures 7a and 7b. The S_BET_ for the Zr_2_AlC MAX phase was calculated to be 1.79 m^2^/g, and the pore volume was 40.4 nm. The pore size was 14.52 nm (desorption average pore diameter (4V/A)).

On the other hand, the S_BET_ of the Zr_2_Al-GNS MAX phase ceramic was increased approximately to 30% from the S_BET_ Zr_2_AlC phase value to be 2.5 m^2^/g with a pore volume of 40.4 nm. The pore size was 13.39 nm (desorption average pore diameter (4V/A)). From the result above, it was found that the S_BET_ of the Z_r2_Al-GNS MAX phase is higher than the S_BET_ of the Zr_2_AlC MAX phase, enhancing the active sites on its surface with well layers’ distribution and similar pore volume, but the average pore size of the Zr_2_Al-GNS MAX phase ceramic is lower than the pore size of the Zr_2_AlC MAX phase due to using nanographene sheet in the structural framework of the Zr_2_Al-GNS MAX phase, increasing the S_BET_, as well as reducing the porosity.

According to the classification of physisorption [57,58], the adsorption/desorption isotherms of the prepared Zr_2_AlC and Zr_2_Al-GNS MAX phases ceramic, as illustrated in Figures 6a and 6b, were assigned a type of III isotherm. In this case, a linear physisorption isotherm curve with no convexity appeared. The adsorbent-adsorbate interactions are weak, and the adsorbed molecules cluster around the most favorable places on the surface of a nonporous or macroporous solid. The structure of the Zr_2_AlC MAX phase describes this. Furthermore, the hysteresis loop is a type of H4 commonly found in micromesoporous carbon materials [57,59,60]. The hysteresis loop of the Zr_2_Al-GNS MAX phase ceramic is very narrow compared with the hysteresis loop of the Zr_2_AlC MAX phase ceramic, which means that the Zr_2_AlC MAX phase contained more random and large pores sizes, as displayed in Figure 6a. The BJH desorption pore size distribution of the Zr_2_AlC MAX and Zr_2_Al-GNS MAX phases was also investigated, as shown in Figures 7a and 7b. According to Figure 7a, the pore size distribution of the Zr_2_Al-GNS MAX phase ceramic is a multilayer with a well-distributed pore size diameter and a more compatible structure than the Zr_2_AlC MAX phase ceramic. In contrast, the pore size distribution was disordered, as shown in Figure 7b. Therefore, using nanosheet graphene in the structure of the Zr_2_Al-GNS MAX phase ceramic improved the S_BET_ with well pore size distribution.

## 5. Electrical properties for potential applications

The electrical properties of the synthesized MAX phases were studied by measuring a group of parameters, as shown in the supplementary material Table S2. These parameters can be classified into two types, such as the primary parameter (L, C & R) and the secondary parameter (D, Q & q). The impedance (Z, ohms (Ω)) can be defined as the total of alternating current oppositions (capacitive reactance, inductive reactance, and resistance), depending on the circuit’s components and the frequency of the applied current [61]. Resistance (R), reactance (X), conductance (G), susceptance (B), quality factor (Q), and dissipation factor (D) are quantities factors that are related to the impedance parameter. At a given frequency (ω), pure element impedance and admittance are real quantities for resistors and pure imaginary for inductors and capacitors, respectively [62]. As an A vector, impedance (Z) is a combination of resistance (R) and reactance (X). When an AC voltage is put across the terminals of an alternating current circuit. The impedance is a complex quantity made up of real (in phase with voltage) and reactive components (90° out of phase with voltage). The impedance can be determined by the following Equation, Equation 5 [61]:


Eq. (5) $$|Z|=\sqrt{R^{2}+X^{2}}$$[
61]

where Z is the impedance in ohms, R is resistance, and X is reactance, the imaginary part of Z in ohms, respectively.

The quality factor (Q) is the percentage of stored energy in a circuit (in C and L) that is lost energy due to resistance (R). The factor of dissipation (D) and quality factor is measured as components “purity”, or whether it is ideal or contains resistance or even reactance. The dissipation factor, D, is the ratio of the real to imaginary parts of impedance, or admittance. As shown in Equation 6, the Q (the quality factor) is the reciprocal of this ratio(63).


Eq. (6) $$\text{D}={\text{R}}_{\text{S}}/{\text{X}}_{\text{S}}={\text{G}}_{\text{P}}/\beta_{\text{P}}=1/\text{Q}$$[
63]

where D is the dissipation factor, R is the resistance in the series circuit in ohms, and X_S_ is the reactance, the imaginary part of Z in ohms. G_P_ is the conductance in a parallel circuit in S, B_P_ is the susceptance in a parallel circuit in Siemen, and Q is the quality factor, respectively. Resistance (R) is an electrical property that prevents current from flowing across a circuit when voltage is applied. Ohm’s law defines resistance as a voltage divided by current (for DC circuits). It is the in-phase or “real” component of impedance in AC circuits. The units of measurement are ohms (Ω) [61].

### 5.1. Effect of the voltage–current on the synthesis ceramic MAX phases

**Figure 8:**
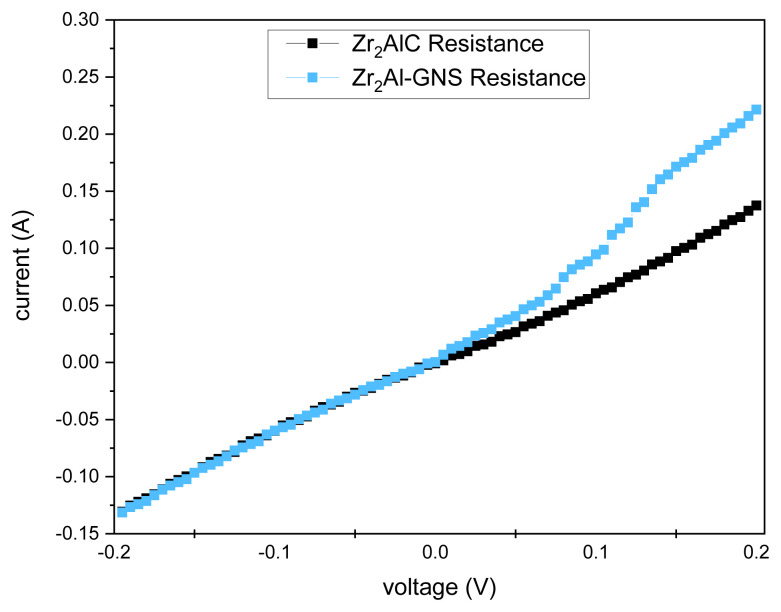
The voltage-current curve for Zr_2_AlC and Zr_2_Al-GNS MAX phases.

The voltage–current relationship was measured for the Zr_2_AlC and the Zr_2_Al-GNS MAX phases, as illustrated in Figure 8. Figure 8 shows a shift in the electrical resistance between the Zr_2_AlC and the Zr_2_Al-GNS MAX phases. The I-V curve corresponded to the synthesized MAX phases in the negative region. In contrast, after the origin point (positive region), the electric resistance (I-V curve) for Zr_2_Al-GNS was increased, improving the electrical resistance in an excellent state. Therefore, the presence of the GNS in the synthesized MAX phase enhanced the electrical resistance significantly. The resistor is a component that represents a linear relationship between voltage and current as dictated by Ohm’s law. The graph of the I-V curve of Ohm’s law equation must be a straight line passing through the origin. Oscillators, memory devices, frequency multipliers, mixers, and other electronic devices and circuits frequently used negative differential resistance (NDR) devices and circuits [53,54,64,65].

### 5.2. Precision LCR meter

**Figure 9:**
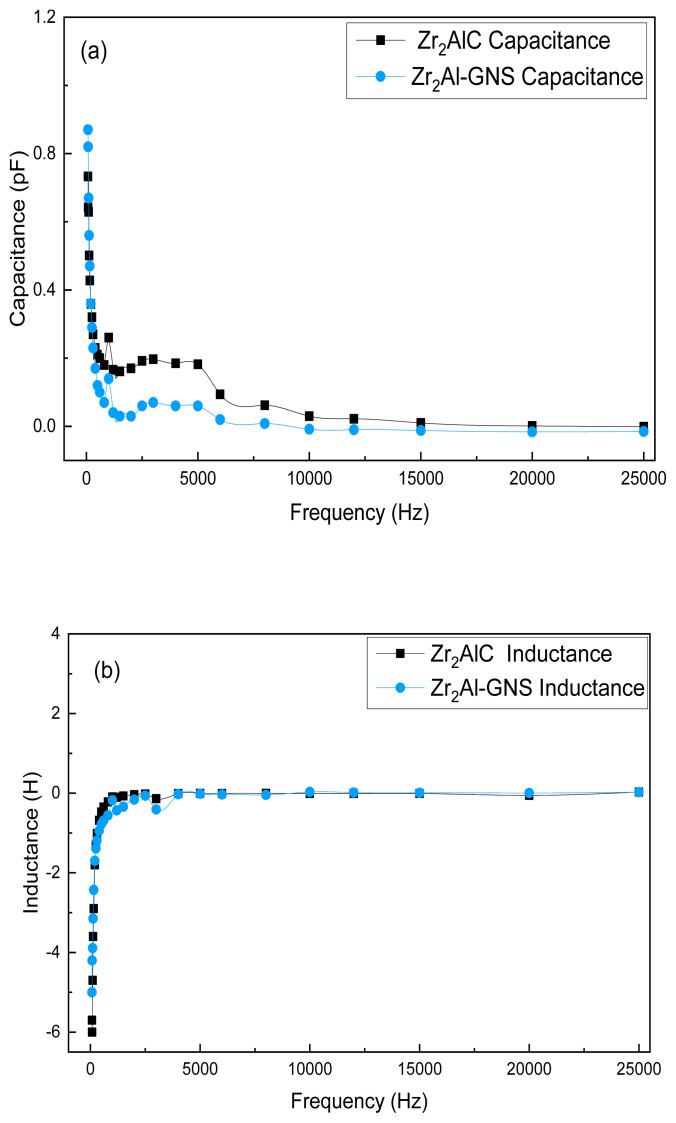
(a–h) (a) Capacitance of Zr_2_AlC phase and Zr_2_Al-GNS phase, (b) inductance of Zr_2_AlC phase plus Zr_2_Al-GNS phase, (c) conductance of Zr_2_AlC phase plus Zr_2_Al-GNS phase, (d) resistance of Zr_2_AlC phase and Zr_2_Al-GNS phase, (e) admittance of Zr_2_AlC phase plus Zr_2_Al-GNS phase, (f) impedance of Zr_2_AlC phase and Zr_2_Al-GNS phase, (g) phase angle of Zr_2_AlC phase and Zr_2_Al-GNS phase, (h) susceptance of Zr_2_AlC phase and Zr_2_Al-GNS phase, (i) (quality factor of Zr_2_AlC phase and Zr_2_Al-GNS phase, (k) dissipation factor of Zr_2_AlC phase and Zr_2_Al-GNS phase, and (l) reactance of Zr_2_AlC phase and Zr_2_Al-GNS phase.

A Precision LCR meter was also utilized to evaluate the electrical properties of the Zr_2_AlC and Zr_2_Al-GNS MAX phases, and the results are illustrated in Figure 9a–9l. The frequency-capacitance relationship shows that the capacitance of the Zr_2_Al-GNS MAX phase ceramic is greater than the capacitance of the Zr_2_AlC phase over a wide frequency range, as shown in Figure 9a. The capacitance of the Zr_2_Al-GNS and Zr_2_AlC MAX phases at a frequency of 75 Hz was 0.87 PF and 0.733 PF, respectively. For more tests, the frequency-inductance relationship between the Zr_2_AlC and the Zr_2_Al-GNS MAX phases was also studied. The inductance changes become positive at the following frequencies, including 10, 15, 20, and 25 kHz for the Zr_2_Al-GNS MAX phase, as shown in Figure 9b. This is because the GNS superiorly enhances the inductance in the structure of the Zr_2_Al-GNS MAX phase. For more details, the prepared MAX phase has a high specific surface area offering tightly bonded atoms in the MAX phase structure, which can improve the inductance property [66]. The frequency-conductance relationship shapes of the Zr_2_AlC and Zr_2_Al-GNS MAX phases were also investigated. According to Figure 9c, the conductance of the Zr_2_AlC MAX phases was decreased. In contrast, the conductance of the Zr_2_Al-GNS MAX phase ceramic was greatly increased at low frequency, indicating that electrons can retain their energy very well inside the Zr_2_Al-GNS MAX phase ceramic with no energy loss. At a frequency of 25,000 Hz, the conductance of the Zr_2_AlC and Zr_2_Al-GNS MAX phases is 10.9 S and 8.4 S, respectively.

Interestingly, the resistance of GNS is low at room temperature due to the electrons’ ability to pass through GNS easily, as well as high-speed electron mobility at room temperature [67,68]. On the other hand, at high frequencies, the resistance increased, and the conductance decreased because of the joint effect of the motion of electrons and holes (*e^−^/h^+^*) pairs. Thus, the low conductance value is possibly due to the low mobility of the electrons and holes in the structure of the MAX phase [69,70].

The frequency–resistance relationship between the Zr_2_AlC and Zr_2_Al-GNS MAX phases was also investigated, as shown in Figure 9d. According to Figure 9d, the resistance of the Zr_2_AlC MAX phase was increased while the resistance of the Zr_2_Al-GNS ceramic MAX phase was greatly reduced at low frequency, resulting in 1.4 M Ω and 1.19 M Ω, respectively, at 75 Hz. Additionally, the frequency–admittance relationship of the Zr_2_AlC and the Zr_2_Al-GNS MAX phases was carried out, and the results are depicted in Figure 9e. At low frequencies, the admittance of the Zr_2_AlC MAX phase was decreased while the admittance of the Zr_2_Al-GNS MAX phase ceramic was increased, as shown in Figure 9e. The admittance of Zr_2_AlC and Zr_2_Al-GNS MAX phases at a frequency of 25,000 H was 14.23 μS and 8.8 μS, respectively.

The frequency-impedance relationship of the Zr_2_AlC and Zr_2_Al-GNS MAX phases was also studied over a wide impedance range, as shown in Figure 9f. According to Figure 9f, the impedance of the Zr_2_Al-GNS MAX phase ceramic is higher than that of the Zr_2_AlC phase over a wide range of values. The impedance of the Zr_2_AlC and the Zr_2_Al-GNS MAX phases were 1.86 M Ω and 1.5 M Ω, respectively, at a frequency of 75 Hz. The impedance was measured by an LCR meter and depends on Equation 5 [63,71].

As shown in Figure 9g, the frequency–phase angle relationship of the Zr_2_AlC and Zr_2_Al-GNS MAX phases was investigated. It shows that the phase angle of the Zr_2_Al-GNS MAX phase ceramic is higher than the phase angle of the Zr_2_AlC MAX phase at a frequency of 25,000 Hz. Zr_2_AlC and Zr_2_Al-GNS MAX phase ceramic phase angles were 1.07° and 18°, respectively. The Zr_2_AlC and Zr_2_Al-GNS MAX phases were also investigated in the frequency–susceptance relationship, as shown in Figure 9h. From Figure 9h, the susceptance of the Zr_2_Al-GNS MAX phase ceramic is lower than the Zr_2_AlC MAX phase. The maximum susceptibilities of the Zr_2_AlC MAX phase and the Zr_2_Al-GNS MAX phase ceramic were 2.1 μS and 5.7 μS, respectively, at a frequency of 500 Hz.

According to Figure 9i, the Zr_2_AlC dissipation factor is larger compared with the Zr_2_Al-GNS MAX phase dissipation factor at low frequency, and the dissipation factor was increased with increasing frequency. In contrast, the dissipation factor of the Zr_2_Al-GNS MAX ceramic drops at high frequency. As shown in Figures 9i and 9k, the quality factor is counter to the dissipation factor, which is smaller for the Zr_2_Al-GNS MAX phase compared with that of the Zr_2_AlC MAX phase. As shown in Figure 9l, the reactance was increased for the Zr_2_AlC MAX phase with a low frequency, and the reactance was decreased for high frequency, but the opposite behavior can be seen in the Zr_2_Al-GNS MAX phase ceramic; it was increased with high frequency and decreased with low frequency. In general, the Zr_2_Al-GNS MAX phase ceramic shows high stability for reactance over a wide range of frequencies.

According to the electrical results, the presence of nanographene sheet significantly enhanced the electrical properties compared to using graphite in the synthesis of the ceramic MAX phase. The results above show that the Zr_2_Al-GNS MAX phase ceramic has more conductivity than the Zr_2_AlC MAX phases ceramic. On the other hand, the electrical conductivity of nanographene sheets is greater than that of graphite. This feature may be the most important governing factor in controlling the electrical properties of the samples in this research. The pore size and porosity of the Zr_2_Al-GNS MAX phase ceramic are lower than the Zr_2_AlC MAX phases ceramic, as discussed in the research. Finally, graphene nanosheets form a two-dimensional hexagonal lattice with flat monolayers of closely spaced carbon atoms [72]. The structure of the graphene nanosheet improved the electrical properties of the prepared MAX phase (Zr_2_Al-GNS MAX phase ceramic). All these parameters mentioned above can explain that the Zr_2_Al-GNS MAX phase ceramic significantly enhanced the electrical properties of the prepared ceramic MAX phase compared with the Zr_2_AlC MAX phases ceramic.

## 6. Conclusion

This study was undertaken to design a new Zr_2_Al-GNS-MAX-phase-ceramic-enhanced one-layer nanographene sheet using a simple pressureless sintering method under low temperatures and evaluate the synthesized temperatures, lattice parameters, and purity percentage. The results of this investigation show that the synthesized temperature of the Zr_2_Al-GNS MAX phase ceramic was 1150 °C. The *a* and *c* lattice parameters were determined to be 3.26 Å and 14.40 Å, respectively. While the crystal diameter of the Zr_2_Al-GNS MAX phase ceramic was 24.2 nm. The results of this study indicate that high dense nanolaminated graphene sheet was extended over a high surface area of the Zr_2_Al-GNS MAX phase ceramic enhanced by the high active sites on its surface with well atomic bonding, implying that the nanographene sheet superiorly reinforced the synthesized MAX phase ceramic. The atomic ratios of the synthesized Zr_2_Al-GNS MAX phase ceramic were Zr (28.26% A), Al (12.40% A), and nanographene sheet (59.34% A). The electrical properties show an improvement in the capacitance of the Zr_2_Al-GNS MAX phase ceramic compared with the Zr_2_AlC MAX phase. The results precisely match the 211 MAX phase ceramic, and the 211-type atomic stacking was clearly obtained. The synthesized Zr_2_Al-GNS MAX phase ceramic is anticipated significantly to contribute to electrical applications, thereby addressing global future development in the electrical field.

## Supplementary Information

Figure S1 Illustration of the mixing of the powder (Zr:Al: GNS) in the vacuumed mill with no agglomeration.

Figure S2 Illustration of the mixing of the powder (Zr:Al: C) in the vacuumed mill with no agglomeration.

Figure S3 Illustration of the phase ratios of (Zr_2_Al-GNS, Zr_2_AlC, Zr_3_Al_2_, Zr_5_Al_3_, and ZrC) at various temperatures (1000 °C, 1150 °C, and 1350 °C).

**Table S1:** The d-spacing and (*hkl*) values were measured using the SAED pattern for the Zr_2_AlC and the Zr_2_Al-GNS.

Zr _2_AlC MAX phase					
Number of layers	1/2r (nm ^−1^)	1/r (nm ^−1^)	r (nm)	d-spacing (Å)	*hkl*
1	5.55	2.77	0.36	3.60	004
2	7.38	3.69	0.27	2.70	010
3	8.59	4.29	0.23	2.32	013
4	12.1	6.05	0.16	1.65	017
5	14.41	7.20	0.13	1.38	001
6	19.16	9.58	0.10	1.04	027
Zr _2_Al-GNS MAX phase					
Number of Layers	1/2r (nm ^−1^)	1/r (nm ^−1^)	r (nm)	d-spacing (Å)	*hkl*
1	7.30	3.65	0.27	2.73	011
2	8.54	4.27	0.23	2.34	006
3	11.95	5.97	0.16	1.67	017
4	14.08	7.04	0.14	1.41	020
5	16.04	8.02	0.12	1.24	026
6	18.73	9.36	0.10	1.06	011

**Table S2:** The group of electrical parameters of electric properties.

Parameter	Quantity	Unit symbol	Formula
	Impedance	Ohm, Ω	=
R = RS	Resistance, Series Resistance	Ohm, Ω	
	Admittance	Siemen, S (was mho)	= =
θ	phase Angle of impedance	degree, deg or radian, rad	θ = arctant) = - ø
ø	phase Angle of Admittance	degree or radian	ø = arctant) = -θ
x	Reactance, Imaginary part of Z	Ohm, Ω	x = 2 f L, 2 f= ω
F	Frequency	henry, H	
L	Inductance	henry, H	
Q	Quality factor	none	Q = = =
D	Dissipation factor	none	D = = =
G	Conductance	Siemen, S	G =
B	Susceptance	Siemen, S	B = 2f C

## Figures and Tables

**Table 1:** The crystallinity phase percentage includes Zr_2_AlC and Zr_2_Al-GNS phases and impurities.

Sample	Zr_2_AlC 1000°C	Zr_2_Al-GNS 1000 °C	Zr_2_AlC1150 °C	Zr_2_Al-GNS 1150 °C	Zr_2_AlC 1300 °C	Zr_2_Al-GNS 1300 °C
Zr_2_AlC wt%	36.4	38.0	40.4	49.0	25.2	29.9
AL_3_Zr_2_ wt%	25.8	25.3	24.7	11.4	14.8	14.5
AL_3_Zr_5_ wt%	20.6	19.9	25.1	31.1	29.5	25.8
ZrC wt%	17.2	16.8	9.8	8.5	30.4	29.8

**Table 2:** The crystallite diameters of the prepared samples at different temperatures are calculated by the Debye-Scherrer equation.

Compound	Temperature	Crystal diameter (nm)
Zr_2_AlC	1000 °C	19.15
Zr_2_Al-GNS	1000 °C	30.79
Zr_2_AlC	1150 °C	10.33
Zr_2_Al-GNS	1150 °C	24.20
Zr_2_AlC	1300 °C	27.05
Zr_2_Al-GNS	1300 °C	14.43

**Table 3:** The lattice parameters compared with previous studies.

Zr_2_Al-GNS ceramic MAX phase	a (Å)	b (Å)	c (Å)	c/a))
Calculated [3]	3.31	3.31	14.63	4.41
Calculated XRD [29]	3.32	3.32	14.57	4.38
Calculated SAED [29]	3.3	3.3	14.6	4.42
Calculated [49]	3.25	3.25	14.5	4.46
Calculated [11]	3.21	3.21	14.24	4.44
Calculated [50]	3.33	3.33	14.60	4.41
Calculated [51]	3.31	3.31	14.60	4.40
This study	3.26	3.26	14.40	4.41

**Table 4:** The EDX analysis illustrated the weight and atomic percentage of the Zr_2_AlC and the Zr_2_Al-GNS MAX phases.

Element	wt%-Zr_2_AlC	A%-Zr_2_AlC	wt%-Zr_2_Al-GNS	A%-Zr_2_Al-GNS
C	19.66	59.34	22.26	58.66
Al	9.23	12.4	17.39	20.4
Zr	71.11	28.26	60.35	20.94
