# Supplementary figures and images for: Synthesizinge a novel Zr2Al-GNS MAX phase ceramic with superior electrical properties using pressureless sintering technique
Source: Turk J Chem. 2023 Jul 4;47(4):763–81. doi: 10.55730/1300-0527.3577 (PMC10760547; doi:10.55730/1300-0527.3577)

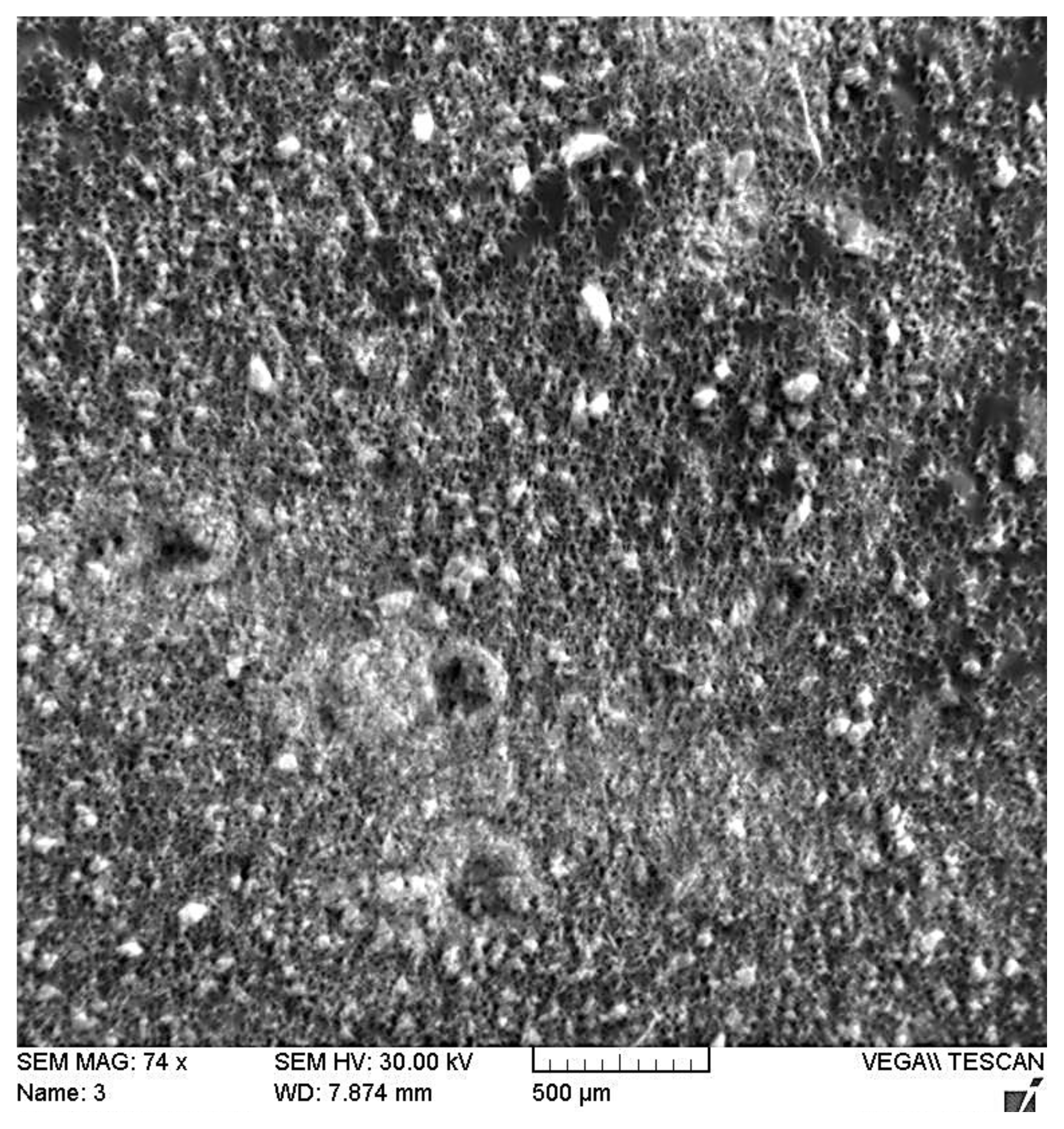

Supplement: Figure S1 — Illustration of the mixing of the powder (Zr:Al: GNS) in the vacuumed mill with no agglomeration. [file turkjchem-47-4-763s1.tif]

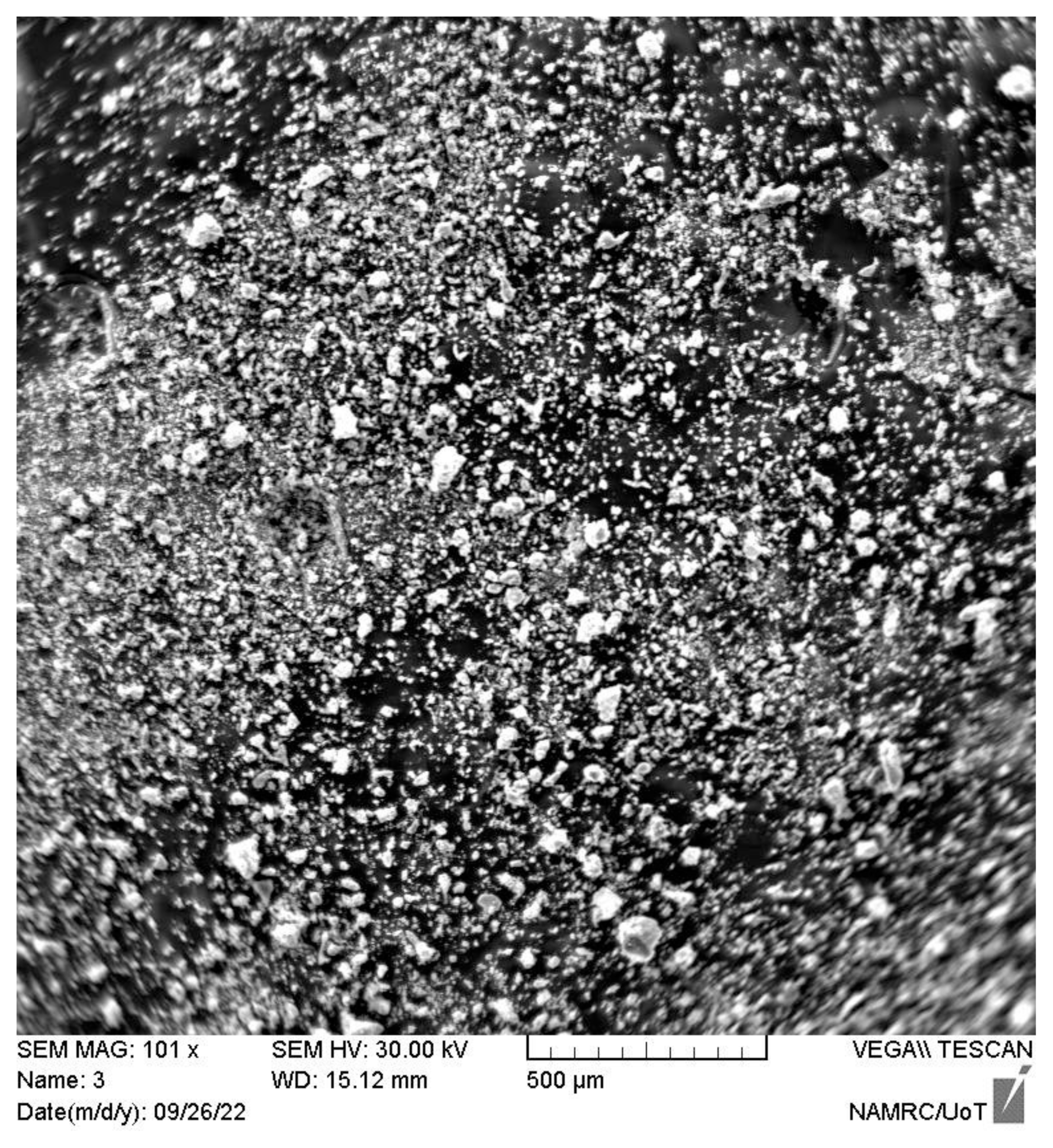

Supplement: Figure S2 — Illustration of the mixing of the powder (Zr:Al: C) in the vacuumed mill with no agglomeration. [file turkjchem-47-4-763s2.tif]

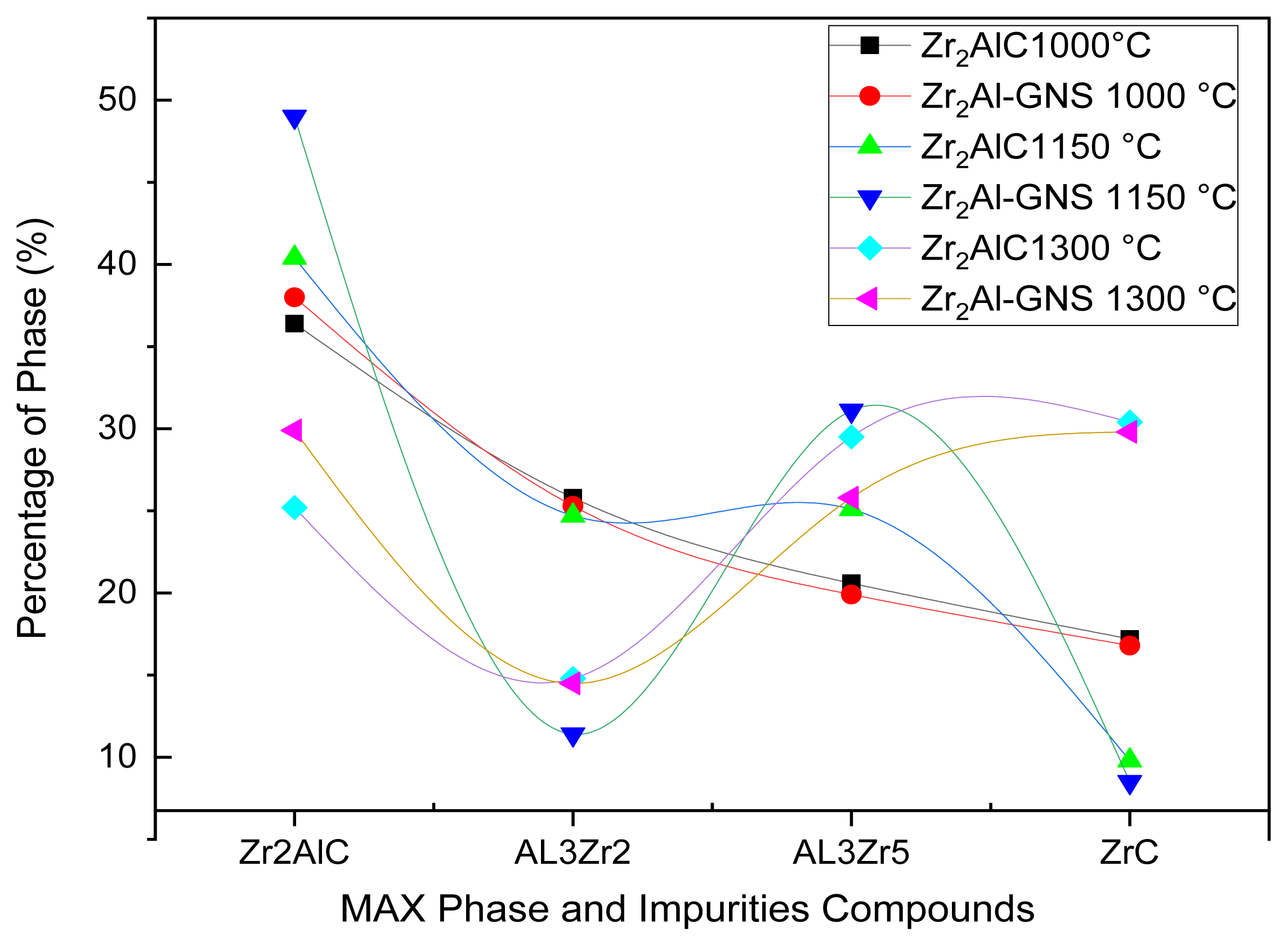

Supplement: Figure S3 — Illustration of the phase ratios of (Zr2Al-GNS, Zr2AlC, Zr3Al2, Zr5Al3, and ZrC) at various temperatures (1000 °C, 1150 °C, and 1350 °C). [file turkjchem-47-4-763s3.tif]
